# Aspects of a Distinct Cytotoxicity of Selenium Salts and Organic Selenides in Living Cells with Possible Implications for Drug Design

**DOI:** 10.3390/molecules200813894

**Published:** 2015-07-31

**Authors:** Ethiene Castellucci Estevam, Karolina Witek, Lisa Faulstich, Muhammad Jawad Nasim, Gniewomir Latacz, Enrique Domínguez-Álvarez, Katarzyna Kieć-Kononowicz, Marilene Demasi, Jadwiga Handzlik, Claus Jacob

**Affiliations:** 1 Bioorganic Chemistry, Department of Pharmacy, Saarland University, Campus B2 1, Saarbruecken D-66123, Germany; E-Mails: ethiene.castellucciestevam@uni-saarland.de (E.C.E.); lisa.faulstich@uni-saarland.de (L.F.); jawad.nasim@uni-saarland.de (M.J.N.); 2 Department of Technology and Biotechnology of Drugs, Faculty of Pharmacy, Jagiellonian University-Medical College, ul. Medyczna 9, Cracow 30-688, Poland; E-Mails: karolawitek.poczta@interia.pl (K.W.); glatacz@cm-uj.krakow.pl (G.L.); enrique.dominguez.alvarez@uj.edu.pl (E.D.-Á.); mfkonono@cyf-kr.edu.pl (K.K.-K.); 3 Laboratório de Bioquímica e Biofísica, Instituto Butantan, São Paulo 05503-001, Brazil; E-Mail: marimasi@butantan.gov.br

**Keywords:** cellular thiolstat, MRSA, proteasome, redox modulation, resistant bacteria, ROS, selenium, tellurium, yeast

## Abstract

Selenium is traditionally considered as an antioxidant element and selenium compounds are often discussed in the context of chemoprevention and therapy. Recent studies, however, have revealed a rather more colorful and diverse biological action of selenium-based compounds, including the modulation of the intracellular redox homeostasis and an often selective interference with regulatory cellular pathways. Our basic activity and mode of action studies with simple selenium and tellurium salts in different strains of *Staphylococcus aureus* (MRSA) and *Saccharomyces cerevisiae* indicate that such compounds are sometimes not particularly toxic on their own, yet enhance the antibacterial potential of known antibiotics, possibly via the bioreductive formation of insoluble elemental deposits. Whilst the selenium and tellurium compounds tested do not necessarily act via the generation of Reactive Oxygen Species (ROS), they seem to interfere with various cellular pathways, including a possible inhibition of the proteasome and hindrance of DNA repair. Here, organic selenides are considerably more active compared to simple salts. The interference of selenium (and tellurium) compounds with multiple targets could provide new avenues for the development of effective antibiotic and anticancer agents which may go well beyond the traditional notion of selenium as a simple antioxidant.

## 1. Introduction

Selenium as an element in general, and selenium salts and organic selenium compounds in particular, are traditionally considered as good antioxidants, as scavengers of free radicals and other Reactive Oxygen Species (ROS) [1,2]. Such compounds may be used in nutrition and therapy as chemopreventive and perhaps even as anticancer agents. Indeed, various selenium-based preparations, such as selenomethionine and sodium selenite (Na_2_SeO_3_), are sold freely in many pharmacies and supermarkets as food supplements, for the prevention of serious diseases, the stimulation of the immune system and also against more or less trivial medical problems, ranging from the common cold to loss of sexual performance and appetite. Recent studies performed by us and others have considered a range of such selenium preparations and uncovered a more sinister, darker side to this apparently antioxidant element [3]. In a yeast model, sodium selenite, for instance, seems to propagate DNA damage, possibly by a direct chemical action or, more indirectly, by inhibition of the relevant repair systems. Similarly, there are reports that an excess uptake of selenium may not prevent but actually promote the formation of certain diseases, possibly even including cancer [4].

Not surprisingly, therefore, a more differentiated view on the biological activity and role of selenium is required, a view which ultimately may open up new perspectives and avenues in drug design and development. Here, the more harmful actions of selenium salts just mentioned at first appear disappointing, yet may also be advantageous if their action could be focused on certain targets, such as bacteria, plasmodia, fungi or cancer cells [5]. Indeed, tellurium salts have long been considered as possible antibiotics, yet the interest in these compounds has declined with the advent of the penicillin era [6]. Nonetheless, with the emergence of multi-resistant bacteria, such as methicillin resistant *Staphylococcus aureus* (MRSA), such older remedies currently experience a certain renaissance in research and development.

The main aim of this study has therefore been to “revisit” of the antibiotic activity of certain selenium and tellurium salts and organic compounds, this time in particular against resistant strains of *S. aureus*, and in combination with known antibiotics. At the same time, the study has tried to uncover some basic aspects of the possible mode(s) of action underlying the (cyto-)toxic activities observed, with a focus on redox regulation and interference with key cellular events.

## 2. Results and Discussion

**Figure 1:**
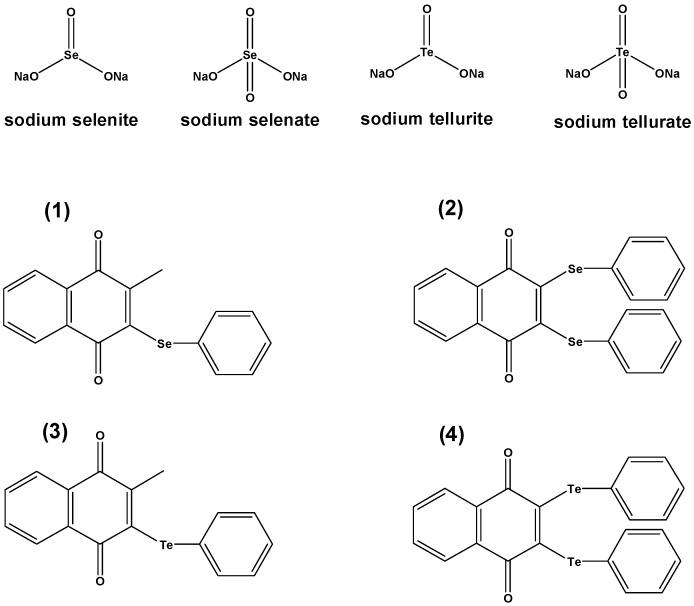
Chemical structures of the various selenium and tellurium compounds employed as part of this study. It should be noted that the salts (**top row**) used are highly polar and often charged, oxidizing compounds with a specific reactivity. Compounds **1**–**4**, in contrast, have been developed over the years as multicenter catalytic sensor/effector agents [8,9].

As part of this study, we have therefore turned our attention to redox active, often catalytic selenium compounds in order to explore their potential action against microorganisms, and also to investigate how such compounds may impact on a living cell. The chemical structures of the compounds used are shown in Figure 1, and these compounds are either available commercially or can be synthesized according to known literature methods [7].

To our great surprise we have found that some simple selenium and tellurium salts act in concert with known antibiotics, hence apparently “sensitizing” such bacteria—which include strains of MRSA—against conventional antibiotics, possibly by inferring with the protease machinery/proteasome. Since such salts are not optimized for a particular biological activity and are also unlikely to penetrate the cell easily, the concentrations required for action were high. At the same time, the formation of red and black deposits could be observed in the case of certain selenium and tellurium salts, respectively. We have therefore subsequently turned our attention towards organic selenium (and tellurium) compounds, and as expected, these compounds show a higher activity. These findings will now be presented and discussed in more detail.

### 2.1. Antimicrobial Activity of Selenium Salts

Since selenium salts, such as Na_2_SeO_3_, nowadays are used widely in form of food supplements, we have first investigated the potential antimicrobial activity of such simple selenium salts and compared this activity to the one of their sulfur and tellurium analogues. This comparison is reasonable as tellurite has long been considered as possible antibiotic and sulfite in fact is used extensively as antimicrobial agent to preserve fruits and vegetable nuts. At the high concentrations used, however, neither selenite or selenate, nor any of the other salts, showed any notable activity against *S. aureus* reference strain ATCC 25923 or multidrug resistant MRSA HEMSA 5 or MRSA HEMSA 5M strains. Indeed, it appears that the MIC values against those bacteria in most instances are well above 1 mM, which is much higher than those of conventional effective antibiotics (Table 1).

**Table 1:** MIC values (in μM) of selected chalcogen salts against three strains of *S. aureus*. Whilst these salts are clearly not active against any of the strains at pharmaceutically relevant concentrations, conventional antibiotics show some activity, which is reduced significantly in the case of the two resistant strains.

Compound		MIC Values (μM) in *S. aureus* Strains		
		ATCC 25923	MRSA HEMSA 5	MRSA HEMSA 5M
**Salt**	**Na_2_TeO_3_**	1000	>2000	>2000
	**Na_2_SeO_3_**	>2000	>2000	>2000
	**Na_2_SeO_4_**	>2000	>2000	>2000
	**Na_2_SO_3_**	>2000	>2000	>2000
	**Na_2_SO_4_**	>2000	>2000	>2000
**Organic**	**1**	31.25	125	500
	**2**	250	500	>1000
	**3**	62.5	62.5	>1000
	**4**	31.25	31.25	125
**Antibiotic**	**Oxacillin**	0.45	340	5400
	**Cloxacillin**	0.42	160	1300
	**Ampicillin/sulbactam**	0.54	200	200
	**Ciprofloxacin**	0.38	14	28
	**Neomycin**	1.0	240	240

This implies, of course, that neither the selenium nor the tellurium or sulfur salts used as part of this study on their own could be employed as antibiotics in practice. Similar results have been obtained against *Saccharomyces cerevisiae*, where neither selenite, selenate nor tellurite showed any significant activity against this type of fungus [3]. It should be noted that tellurate (Na_2_TeO_4_) was also considered, yet could not be tested reliably because of solubility issues in the buffers used which resulted in irreproducible results.

This rather disappointing finding is not entirely unexpected: such simple, highly polar salts are not optimized for antibiotic action and their ability to cross cell membranes is limited. Nonetheless, the interactions of such salts with biomolecules, especially cysteine containing proteins, are well documented *in vitro*, and one would be tempted to expect at least some impact on the bacterial, fungal or mammalian cell. Based on our previous studies in this field, one may speculate, for instance, that some of these compounds, particularly Na_2_SeO_3_, could “weaken” the cell by promoting oxidative stress and damage to DNA or by affecting the cellular thiolstat [10,11].

In the case of the organic selenium and tellurium compounds **1**–**4**, a slight antibacterial activity could be observed when these compounds were used in micromolar concentrations. While this activity often was distinctively lower than that of the reference antibiotics used, it was much higher when compared to the inorganic salts—and was also rather competitive in the case of the drug resistant strains. The tellurium compounds **3** and **4**, in particular, showed some promising activity against the multidrug resistant strain HEMSA-5. The *bis*-phenyltellanyl derivative **4** was even able to inhibit the growth of both MDR MRSA strains (HEMSA 5 and extremely resistant HEMSA 5M) at a dose significantly lower than that of most antibiotics tested (excluding ciprofloxacin, see Table 1).

Since it appears that the selenium and tellurium compounds act differently, probably on different targets when compared to the conventional antibiotics, we have posed the question if they may not be able to also act synergistically, *i.e.*, in concert with conventional agents. We have therefore investigated both, the organic compounds **1**–**4** and some of the salts in combination with known antibiotic agents, such as oxacillin, cloxacillin, ampicillin/sulbactam, ciprofloxacin and neomycin, and indeed observed a significant enhancement in the antibacterial activity of such traditional antibiotics, even against some of the strains of MRSA (Table 2). Notably, whilst the inorganic salts often enhanced the efficiency of the antibiotics used, the organic compounds **1**–**4** were not particularly useful in those combination assays, bearing in mind, of course, that compounds **3** and **4** are fairly potent antibiotics on their own (see above). Particularly noteworthy, for instance, is the influence of tellurite (Na_2_TeO_3_), which at a concentration of just 500 µM enhances the toxicity of oxacillin and cloxacillin against MRSA HEMSA 5M by more than 1000-fold (Table 2). Notably, Na_2_TeO_3_ does not seem to exhibit any synergistic effects against the reference strain *S. aureus* ATTC 25923.

**Table 2:** Ability of the chalcogen compounds tested to enhance the antibacterial activity of selected antibiotics against *S. aureus* strains at a chalcogen compound concentration of 500 µM. Results are expressed as the quotient of the MIC value of antibiotics in the absence to that in the presence of the corresponding chalcogen compound.

Antibiotic	Strain of *S. aureus*	Antibiotic Efficacy Enhancement $\left(\frac{MIC_{Ant}}{MIC_{Ant+Comp}}\right)$					
		1–4	Na_2_TeO_3_	Na_2_SeO_3_	Na_2_SeO_4_	Na_2_SO_3_	Na_2_SO_4_
**Oxacillin**	ATCC 25923	NE	NE *	4	4	NE	NE
	MRSA HEMSA 5	NE	16	NE	NE	NE	NE
	MRSA HEMSA 5M	NE	1024	NE	NE	NE	NE
**Cloxacillin**	ATCC 25923	NE	2 *	4	4	NE	NE
	MRSA HEMSA 5	NE	256	NE	NE	32	32
	MRSA HEMSA 5M	NE	≥1024	NE	NE	NE	NE
**Ampicillin/Sulbactam**	ATCC 25923	NE	4 *	2	2	NE	NE
	MRSA HEMSA 5	NE	8	NE	NE	4	4
	MRSA HEMSA 5M	NE	8	NE	NE	NE	NE
**Ciprofloxacin**	ATCC 25923	NE	NE *	NE	NE	NE	NE
	MRSA HEMSA 5	NE	NE	NE	NE	NE	NE
	MRSA HEMSA 5M	NE	NE	NE	NE	NE	NE
**Neomycin**	ATCC 25923	NE	2 *	NE	NE	NE	NE
	MRSA HEMSA 5	NE	4–8	8	NE	NE	NE
	MRSA HEMSA 5M	NE	4	NE	NE	NE	NE

NE: No enhancement observed; * Tellurite was evaluated at a concentration of 250 µM (*i.e.*, at one quarter of direct MIC in ATCC25923).

These findings are rather interesting, as they indicate a certain “re-sensitization” of the drug resistant strains against classic antibiotics in the presence of certain selenium, and possibly also of tellurium or sulfur compounds. Tellurite, for instance promotes the activity of β-lactam antibiotics oxacillin and cloxacillin against both strains of MRSA studied, reducing the MIC values for these antibiotics from well over 100 µM in the case of HEMSA 5 and over 1000 µM in the case of HEMSA 5M to acceptable values in the sub- or low micromolar range, respectively. Furthermore, tellurite increases the action of ampicillin combined with sulbactam or of the aminoglycoside antibiotic neomycin against multidrug resistant MRSA strains (Table 2).

This finding in itself obviously is exceptionally attractive from the perspective of future drug combination therapies, which to a large extent will rely on this kind of re-sensitization of resistant strains. It also agrees with a very recent report in the literature, which shows that nanomolar concentrations of Na_2_TeO_3_ can enhance the toxic effects of the cephalosporin antibiotic cefotaxime against *Escherichia coli* [12]. In this study, the enhancement by Na_2_TeO_3_ appeared to be due to a tellurite-induced oxidative stress, and a widespread damage to DNA and to proteins, possible mode(s) of action which will be considered in more detail as part of the following sections.

Unfortunately, tellurite is also rather toxic in humans, and the two selenium salts are less active, yet also seem to be able to somewhat, up to 4- to 8-fold, promote the toxicity of different antibiotics. Na_2_SeO_3_, in particular, seems to enhance the action of aminoglycosides against multidrug resistant MRSA HEMSA 5, decreasing the effective dose of neomycin required from 240 µM to 30 µM. Bearing in mind that Na_2_SeO_3_ and Na_2_SeO_4_ are considerably less toxic towards humans when compared to their tellurium analogues, such an enhancement of antibiotic action in MRSA strains is rather interesting. The ultimate choice for a potential therapeutic use therefore may well consider selenite and selenate, rather than tellurite, and obviously at concentrations not damaging or even lethal to humans. Incidentally, an interesting activity of sulfite and sulfate—which at first were included in the study as controls—could be demonstrated. The toxicity of cloxacillin against MRSA HEMSA 5, for instance, is enhanced around 32-fold by 500 µM concentrations of either Na_2_SO_3_ or Na_2_SO_4_. Since sulfate, in particular, is non-toxic to humans even when administered at higher concentrations, this finding—and the possible usage of sulfur-based salts as potential “adjuvants” of this β-lactam antibiotic—should also be considered further in earnest.

### 2.2. Cellular Targets of Selenite and Selenate in Bacteria and Yeast

Whilst some of the selenium and tellurium salts studied seem to enhance the antibiotic activity of traditional antibiotics, such as β-lactams and aminoglycosides, none of chalcogen salts employed was able to increase the bactericidal activity of the fluoroquinolone ciprofloxacin. Ciprofloxacin is an inhibitor of bacterial gyrase and hence DNA replication. In contrast, the penicillins interfere with the bacterial cell wall synthesis. It is therefore possible that the synergistic effects observed in the case of the penicillins point toward a particular mode of action of salts such as Na_2_TeO_3_, Na_2_SeO_3_ or Na_2_SeO_4_. We have already mentioned in passing that based on the chemistry and *in vitro* studies of such redox active selenium and tellurium compounds, one would expect a “weakening” of cells exposed to higher concentrations of these agents. Although speculative at this time, it may be possible, for instance, that these salts interfere with the cell membrane or components thereof. They may hinder the cell wall synthesis, perhaps inhibit (cysteine containing) β-lactamases or stress the cell more generally, for instance by the generation of Reactive Oxygen Species (ROS) [13,14]. Indeed, such actions have been associated with selenite and tellurite before [3,12].

**Figure 2:**
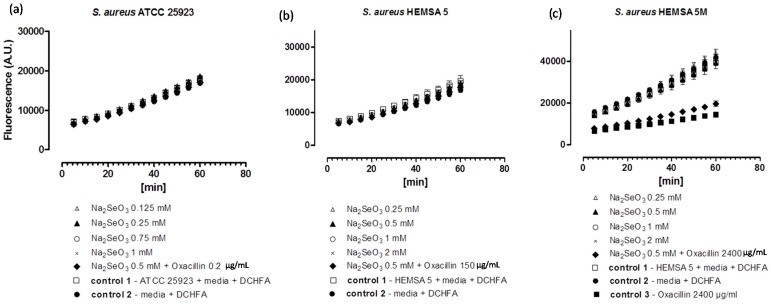
Generation of intracellular ROS by Na_2_SeO_3_ in different strains of *S. aureus*. ATCC 25923 (**a**); HEMSA 5 (**b**) and HEMSA 5M (**c**). Data is shown in terms of fluorescence emitted (in arbitrary units) by (oxidized) DCFA. The latter is generated by the reaction of certain ROS and Reactive Nitrogen Species (RNS) with DCHFA. Different concentrations of Na_2_SeO_3_ were assayed and the influence of the dual presence of the chalcogen salt and an antibiotic (oxacillin) on ROS generation was also evaluated. Values shown represent mean values with *n* = 4. The error bars represent the standard deviation (SD) and statistical significances were calculated using an one-way ANOVA followed by Bonferroni’s multiple comparison test (Figure 2a,b: *p* > 0.05; 2c: *p* < 0.05).

**Figure 3:**
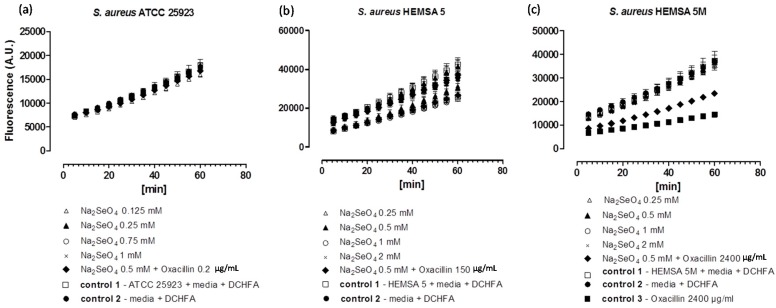
Generation of intracellular ROS by Na_2_SeO_4_ in different strains of *S. aureus*. ATCC 25923 (**a**); HEMSA 5 (**b**) and HEMSA 5M (**c**). See legend of Figure 2 for further details.

**Figure 4:**
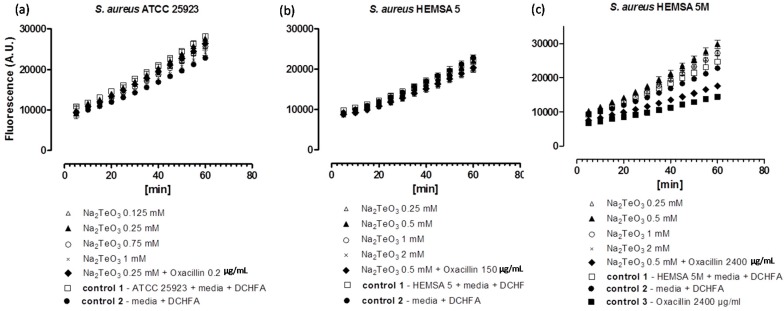
Generation of intracellular ROS by Na_2_TeO_3_ in different strains of *S. aureus*. ATCC 25923 (**a**); HEMSA 5 (**b**) and HEMSA 5M (**c**). See legend of Figure 2 for further details.

To investigate this issue further, we have therefore considered the impact of these chalcogen salts on intracellular ROS levels in *S. aureus* ATCC 25923, HEMSA 5 and HEMSA 5M using 2ʹ,7ʹ-dichlorodihydrofluorescein diacetate (DCHFA) as redox-sensitive fluorescent indicator dye. The results of this crude ROS assay are presented for the different salts, and against different strains of *S. aureus* in Figure 2, Figure 3 and Figure 4.

Despite the fact that some of the salts notably increased the efficiency of some of the antibiotics, none of the inorganic selenium or tellurium salts studied, either on its own or in combination with oxacillin, increased the intracellular levels of ROS significantly. This finding is truly unexpected, as compounds such as selenite are known to generate ROS *in vitro* [15,16,17], are able to modify the thiol redox state and cellular thiolstat, and also have the potential to inhibit enzymes involved in the reduction of oxidative stress. Selenite even promotes DNA strand breaks in yeast, possibly also via a redox mechanism [3,18,19,20]. As already mentioned above, there are also reports that K_2_TeO_3_ causes a rather strong increase in intracellular ROS levels in Gram-negative *E. coli* [21]. Hence, at least in theory, one may have expected an increase in ROS levels for Na_2_TeO_3_ and Na_2_SeO_3_, either by the direct chemical generation of additional ROS, by damaging cellular components subsequently leading to an indirect increase of ROS concentrations, or by the inhibition of ROS removal systems. Still, it seems that at the concentrations used, none of these redox events plays any major role in the bacteria studied here.

Noteworthy, there is also no consistent evidence of any “antioxidant” activity of Na_2_TeO_3_, Na_2_SeO_3_ and Na_2_SeO_4_ in the bacteria investigated. Generally, no significant differences in fluorescence in the presence of the compound and the controls were observed. Only in one case, *i.e.*, when the HEMSA 5M strain was treated with oxacillin (2400 µg/mL) (with or without the presence of the salts examined), could a significant decrease (*p* < 0.005) of fluorescence compared to the control be observed (Figure 2c, Figure 3c and Figure 4c). This observed decrease in fluorescence, however, could be due to the high concentration of oxacillin used in the ROS assay of this strain, which may have influenced the natural conversion of DCHFA into DCFA. Indeed, a specific control (*i.e.*, control 3) has been used in those cases to confirm that oxacillin, and not the selenium or tellurium salts, is probably responsible for this particular observation.

Eventually, one must also emphasize that the DCHFA-assay is widely used yet far from perfect: it does not capture all ROS and its outcome is also affected by the activity of certain cellular esterases (see also Section 3). Furthermore, Kalyanaraman has suggested that the intracellular oxidation of this dye may be mediated to some extent by iron or by cytochrome *c* [22]. Hence any results obtained by this fluorescent assay are rough estimates only and need to be treated with some caution. Whilst the DCHFA assay is still widely accepted as a good measure of intracellular levels of ROS, a more sensible, *i.e.*, sensitive and selective method should be used in future studies to confirm the lack of pro-oxidant activity of the salts tested.

**Figure 5:**
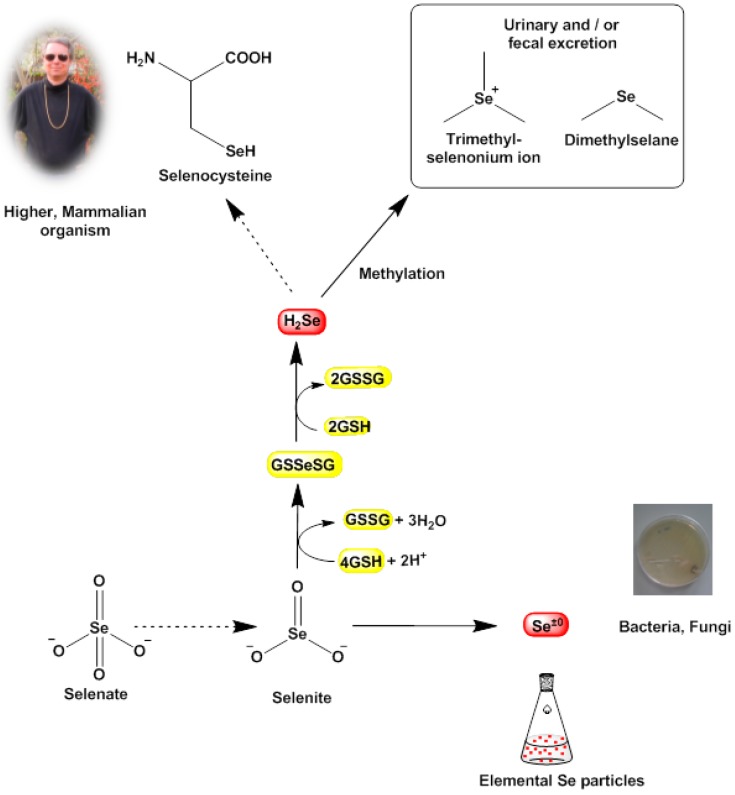
Known bioreductive pathways for SeO_3_^2−^ (and TeO_3_^2−^) in certain bacterial cells. These pathways often terminate in the formation of (solid) elemental selenium and tellurium (nano)particles, which subsequently may cause damage to the cell. In our studies, it appears that selenite (SeO_3_^2−^) and tellurite (TeO_3_^2−^) are also reduced by *S. aureus*, as different reports have observed similar changes in color and the formation of deposits and have linked these physical changes to the formation of selenium and tellurium, respectively. In sharp contrast, mammalian cells tend to by-pass the “zero oxidation state” and form H_2_Se as a valuable raw product for the synthesis of the amino acid selenocysteine, whilst excess selenium is excreted in form of methylated products. Hence the bioreductively generated toxicity of selenium and tellurium (nano)particles is probably more or less specific for lower organisms.

More or less by coincidence, however, we have observed a rather different, perhaps not entirely expected biological redox activity associated with some of these salts, which may well explain some of the findings shown in Table 2 and which may also provide a new angle to the discussion. Upon exposure to tellurite and selenite salts, the strains of bacteria investigated changed color, and in fact generated and then obviously accumulated solid red and black deposits, which based on previous reports appear to consist of elemental selenium and elemental tellurium, respectively. Indeed, a closer look at the literature on bacteria dealing with selenium and tellurium salts shows that this finding is not wholly unanticipated. Many bacteria, including *Halococcus salifodinae BK18*, *Azospirillum brasilense*, *Sulfurospirillum* and *Thauera selenatis* and also some more common ones such as *Pseudomonas*, *Lactobacillus* and *Bacillus cereus* are known to reduce selenite to elemental selenium (nano)particles [23,24,25,26,27,28,29]. *Thauera selenatis* is even able to reduce selenate to form elemental selenium deposits. In fact, different species of *Lactobacillus* are used as a natural source of selenium nanoparticles, for instance to enrich probiotics with selenium [30]. Similarly, bacteria such as *Bacillus beveridgei*, *Rhodobacter capsulatus* and even *E. coli* readily reduce Na_2_TeO_3_ to generate elemental tellurium, usually in form of nanoscopic or microscopic rods and needles [31,32,33]. Such a reactive cascade leading to elemental selenium or tellurium is shown in Figure 5. It should be noted that to the best of our knowledge, there have been no reports so far that *Staphylococcus* converts selenite or tellurite to elemental selenium or tellurium, respectively.

Indeed, the elemental precipitates formed not only stain the bacterial cells red or black, those particles also exert a massive mechanical stress on the bacterial cell which may ultimately cause cell death via a combination of mechanical stresses and unfavorable cellular responses toward them. Eventually, the bioreductive formation of these solid deposits in the bacterial cell may weaken the cell and hence may also explain—at least in part—the notable efficiency of traditional antibiotics in such compromised cells. This process may also contribute to the selective action of such simple chalcogen salts, as mammalian cells do not reduce selenite to elemental selenium. Here, selenite is mostly “sequestered” chemically by glutathione (GSH) in form of glutathione selenotrisulfide (GSSeSG). The latter is reduced further to hydrogen selenide (H_2_Se) which either serves as a selenium source for the synthesis of selenocysteine or is methylated to dimethylselenide (CH_3_SeCH_3_) and ionic trimethylselenonium ((CH_3_)_3_Se^+^) and excreted (Figure 5) [34,35]. In any case, mammalian cells normally do not form solid selenium or tellurium particles and hence are not affected by the mechanical stresses and toxicity caused by such particles [36].

Since the reduction of salts such as Na_2_SeO_3_ often proceeds spontaneously in the presence of thiols (such as GSH), another mode of (cytotoxic) action has been discussed for various selenium and tellurium compounds [37]. The underlying chemical mechanism includes the—probably spontaneous and more or less random—reaction of such compounds with thiol groups in proteins. Such reactions result in various posttranslational oxidative modifications which may also impact on the function and activity of the proteins and enzymes affected. Since many cysteine proteins and enzymes are involved in pivotal cellular processes (metabolism, signaling, maintenance of redox homeostasis *etc.*), and hence have recently been subsumed under the notion of the “cellular thiolstat”, a modification of their cysteine residues and thus function and activity may have serious consequences for the cell [10].

**Figure 6:**
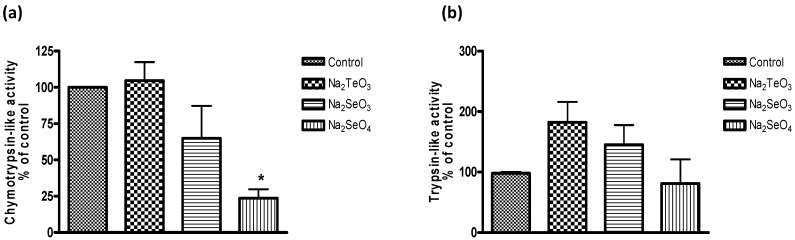
Inhibition of the proteasome in intact cells of *S. cerevisiae* (chymotrypsin-like activity (**a**) and trypsin-like activity (**b**)). Data was obtained from cell extracts after 3 h incubation in the presence of 1000 µM of tellurium and selenium salts. Values represent means with SD bars from at least three independent experiments, and statistical significances were calculated using a one-way ANOVA followed by Bonferroni’s multiple comparison test. * *p* < 0.05.

As part of our study, we have therefore considered if such compounds also interact with key cellular components or events, especially those which are related to the cellular thiolstat and whose inhibition may be highly detrimental to the cell. As part of this search for potential protein targets, we noticed a significant inhibition of the proteasome in yeast (Figure 6). It should be pointed out that *S. cerevisiae* was used as model as the yeast proteasome can be studied reliably in isolation as well as in the intact cell. Here, Na_2_SeO_3_ and especially Na_2_SeO_4_ were most active. The impact of Na_2_SeO_4_ on proteasome activity was observed at higher micromolar concentrations and, at a concentration of 1 mM, Na_2_SeO_4_ reduced the chymotrypsin-like activity of the yeast proteasome in intact cells significantly, to less than 25%.

Arguably, these concentrations of selenate are high and not relevant physiologically. Still, one needs to bear in mind that these experiments were performed with intact cells, and intracellular concentrations of Na_2_SeO_4_ may be lower. In fact, the known proteasome inhibitor bortezomib, which was used in this part of the study as positive control, failed to inhibit proteasome activity in yeast cells altogether when used at its standard concentration of 1 micromolar. Whilst bortezomib and similar inhibitors penetrate human cells readily, they cannot penetrate wild-type yeast cells due to an inherent impermeability of the cell wall or membranes [38]. For this reason, yeast has been used simply as a model system, and not as a target for therapeutic intervention.

Notably, both selenium salts did not inhibit the trypsin-like activity and Na_2_TeO_3_, often considered as the most active of these chalcogen salts, had no inhibitory effect on the yeast proteasome under the conditions used. Indeed, at closer inspection, it even appears that Na_2_SeO_3_ and Na_2_TeO_3_ may actually stimulate the trypsin-like activity, although those increases are statistically not significant (Figure 6b).

Once more, one may speculate that the compounds employed are able to react with thiol groups in proteins, and therefore may also interfere with and inhibit a range of proteins and enzymes, including parts of the proteasome, either by a direct modification of cysteine residues in components of the proteasome itself or by a widespread modification of proteins which then end up and “overload” the proteasome in a detrimental manner. Indeed, the notion that thiol-selective yet otherwise unspecific (re-)agents may be able to modify a wide range of cellular, redox-sensitive cysteine-proteins, which subsequently not only lose their function and activity, but also end up as “garbage” and overload the proteasome, is highly stimulating and deserves a more detailed consideration in the future.

On the one side, these findings are rather instructive, as the proteasome represents an important target in modern therapy. In eukaryotes, the ubiquitin-proteasome system is the main proteolytic system, whilst in bacteria there are different proteolytic complexes that are directly linked to bacterial virulence [39,40]. Not surprisingly, the proteolytic machinery is being considered as a promising therapeutic target, especially in multi-resistant bacterial species [41]. Here, fellutamide B acts as inhibitor of the *Mycobacterium tuberculosis* proteasome, whilst an inhibitor of the protease ClpXP increases the sensitivity of methycillin-resistant *S. aureus* and *Bacillus anthracis* to antibiotics that act on the bacterial wall (synthesis) and membrane [42,43]. Indeed, such observations on the “re-sensitization” of the drug resistant strains against conventional antibiotics acting on the cell wall synthesis via an inhibition of the proteasome mirror most of the still preliminary findings of our present study [39,40,41,42,43]. It is therefore a highly rewarding task to study the impact of selenium food supplements and organic selenium compounds on different proteolytic systems/proteasomes (e.g., bacteria, fungi, human cancer cells, normal cells) more extensively in the future.

On the other side, the proteasome represents probably just one target for salts such as Na_2_SeO_3_ and Na_2_SeO_4_. There may be many other—yet unidentified—targets, and some of them may also differ between bacterial, fungal and mammalian cells, depending on the specific components of the respective cellular thiolstat and the metabolic processes in action (such as specific bioreductive pathways).

In any case, the observation that simple salts, such as Na_2_SeO_3,_ are able to compromise bacterial cells, even employing some redox driven “nanotechnology”, underlines the considerable interest in redox active and catalytic selenium compounds—some of which form part of our daily nutrition—and their potential uses in medicine.

### 2.3. From Simple Selenium Salts to Redox Modulating Selenium Drugs

Whilst the results obtained so far for Na_2_SeO_3_ and Na_2_SeO_4_ (as well as for Na_2_TeO_3_) are interesting, such compounds are not particularly useful from a pharmaceutical point of view, not least because of the high concentrations required for a significant activity. As already mentioned, we have therefore investigated some of the most interesting findings obtained as part of this study further, this time employing some of our most potent redox modulating selenium and tellurium agents (compounds **1**–**4** in Figure 1) [5,7,44,45]. Since these compounds have been designed as potential therapeutic agents (for instance considering reactivity, stability, pharmacokinetics, Lipinski’s “Rule of Five”, *etc.*), it was hardly surprising to find a pronounced antibacterial activity for the selenium compound **1** against *S. aureus* ATCC25923 with MIC values as low as 31.25 µM. Interestingly, the two tellurium compounds **3** and **4** were even rather active against the MRSA strain HEMSA 5, with MIC values of 62.5 µM and 31.25 µM, respectively. These MIC values are rather low, also when compared to the MIC values listed for some of the conventional antibiotics in Table 1. Compound **4**, which contains two tellurium centers and a quinone moiety, therefore seems to be particularly promising as a potential antibacterial agent, and on its own [46]. 

When considered in combination with traditional antibiotics, such as oxacillin, these compounds did not enhance the activity of the antibiotics used. Yet again, this is hardly surprising. The special bioreductive “chemistry” witnessed in bacteria in the case of salts such as Na_2_SeO_3_, Na_2_SeO_4_ and Na_2_TeO_3_, for instance, is notably absent for compounds **1**–**4**. First and foremost, no color changes were observed when the organoselenium and organotellurium compounds were used. Secondly, there was also no evidence of any deposit formation when the cells were incubated with the organic selenium and tellurium compounds. Hence based on this evidence, it appears that no elemental selenium and tellurium particles similar to the ones observed for the salts were formed and that the kind of mechanical and (bio)chemical stresses discussed for compounds such as Na_2_SeO_3_ and Na_2_TeO_3_ was therefore almost certainly also absent.

Besides simple changes in color, these differences in “chemistry” between the inorganic and organic selenium and tellurium compounds obviously translate into further differences in biochemical activity. The organic compounds employed contain several redox active, catalytic centers and are known to interfere with the intracellular redox homeostasis in human cells. As part of a related study, it has moreover become apparent that such compounds, especially compounds **2** and **4**, are able to interfere with proteasomes in intact cells as well as with isolated human proteasomes, where those two compounds at a concentration of just 1 µM reduce chymotrypsin-like activity by 80% and trypsin-like activity by 90%. We also know from our previous studies that these compounds inhibit mammalian thioredoxin reductase (TrxR) *in vitro*, and at rather low concentrations. The selenium compound **2**, at just 1 µM, reduces TrxR activity to less than 50%, whilst the tellurium compound **3** is even more active, with just 60% of the original TrxR activity remaining when used at 500 nM and less than 20% TrxR activity remaining when used at an 1 µM concentration [47]. There is also evidence that selenium compounds closely related chemically to **1** and **2** interfere with protein synthetic pathways and ultimately protein synthesis [47].

## 3. Experimental Section

### 3.1. Materials, Solvents and Bacteria

#### 3.1.1. Materials

Chemicals, including the various sulfur, selenium and tellurium salts, educts and solvents for the chemical synthesis, and substances used for yeast cell culture were purchased from Sigma-Aldrich (Steinheim, Germany). Sodium selenite (Na_2_SeO_3_) and sodium tellurite (Na_2_TeO_3_) were obtained from Merck KGaA (Darmstadt, Germany). Sodium sulfite (Na_2_SO_3_), sodium sulfate (Na_2_SO_4_), the antibiotics oxacillin, cloxacillin, ampicillin/sulbactam, ciprofloxacin and neomycin and the fluorescent redox indicator dye 2ʹ,7ʹ-dichlorodihydrofluorescein diacetate (DCHFA) were purchased from Sigma-Aldrich (Poznań, Poland). Chemicals were used without further purification unless stated otherwise. Compounds **1**–**4** were synthesized according to established literature procedures and purified. Their analytical data (^1^H-NMR, ^13^C-NMR, mass spectrometry) was in accordance with values reported in the literature [44]. The fluorescent substrates required for measurements of the proteasome activity (suc-LLVY-AMC and z-ARR-AMC) were purchased from Calbiochem (Merck Chemicals GmbH, Schwalbach, Germany).

#### 3.1.2. Solvents

Distilled water (Telmed Destylator DE 8/70, Warsaw, Poland) was used as solvent for the preparation of Mueller-Hinton Broth (MHB) media and all antibiotic stock solutions. MHB medium was sterilized by autoclaving at 121 °C for 15 min. DCHFA stock solution (2 mM) was prepared in pure ethanol (≥99.8%, Avantor Performance Materials Poland, Gliwice, Poland). MilliQ water (resistance ≥ 18 MΩ·cm^−1^) was used for the studies involving *S. cerevisiae*.

#### 3.1.3. Bacterial Strains

Wild-type *S. aureus* American Type Culture Collection ATCC 25923 (oxacillin MIC 0.2 µg/mL) and clinical methicillin resistant MRSA strains HEMSA 5 (oxacillin MIC 150 µg/mL) and MRSA HEMSA 5M (oxacillin MIC 2400 µg/mL) were used as part of this study (Instituto de Higiene e Medicina Tropical, Universidade Nova, Lisbon, Portugal).

### 3.2. Microbiological Activity Assays

The impact of compounds **1**–**4** and inorganic salts was evaluated in three strains of *S. aureus*: the reference strain ATCC 25923 and the methicillin resistant strains MRSA HEMSA 5 and MRSA HEMSA 5M. Minimum inhibitory concentrations (MICs) of oxacillin, cloxacillin, ampicillin/sulbactam, ciprofloxacin and neomycin, as well as MICs of compounds **1**–**4**, Na_2_TeO_3_, Na_2_SeO_3_, Na_2_SeO_4_, Na_2_SO_3_, and Na_2_SO_4_, *i.e.*, the intrinsic antibacterial activity of the compounds, and the ability of the selenium and tellurium compounds to enhance the activity of the above-mentioned antibiotics were assessed by a broth microdilution method in Mueller-Hinton Broth (MHB) according to Clinical and Laboratory Standards Institute (CLSI) recommendations. The MICs were recorded as the lowest concentration of the compound or of the antibiotic inhibiting visible growth of bacteria after a 16 h incubation at 37 °C. In the first step, intrinsic antibacterial activity of each compound was examined. Subsequently, the MICs of oxacillin, cloxacillin, ampicillin/sulbactam, ciprofloxacin and neomycin were determined in the absence and presence of the compounds to investigate any synergistic effects. In order to avoid any significant toxicity of the selenium and tellurium compounds in this step, their concentrations were no greater than a quarter of their respective MIC values. All microbiological assays involving bacteria were performed in at least two repetitions, whilst the studies with yeast were performed in triplicate and on three different occasions.

### 3.3. Determination of Intracellular Levels of Oxidative Stress via the DCHFA Assay

Intracellular levels of ROS were estimated using the fluorescent dye DCHFA. The latter is a non-polar dye which can cross cell membranes and becomes trapped inside cells by deacetylation. The deacetylated form subsequently reacts with certain ROS to produce 2ʹ,7ʹ-dichlorofluorescein (DCFA), the oxidized, fluorescent form of DCHFA. It should be noted that certain ROS, such as the hydroxyl radical (^•^OH) or hydrogen peroxide (H_2_O_2_) react better with DCHFA in this assay compared to other ROS, and that oxidation can also be triggered by certain Reactive Nitrogen Species (RNS) [22,48].

As part of this assay, the reference strain *S. aureus* ATCC 25923 and the clinical strains *S. aureus* MRSA HEMSA 5 and MRSA HEMSA 5M were grown in Mueller-Hinton Broth (MHB) at 37 °C with shaking until reaching an optical density of 0.5 at 600 nm (OD_600_ = 0.5). At this point the bacterial cells were loaded in a 96-well plate and treated with different concentrations of Na_2_TeO_3_, Na_2_SeO_3_ or Na_2_SeO_4_ (0.125, 0.25, 0.75 and 1 mM for *S. aureus* ATCC 25923 and 0.25, 0.5, 1 and 2 mM for *S. aureus* MRSA HEMSA 5 and MRSA HEMSA 5M). ROS levels in bacterial cells were also investigated after adding both an inorganic salt (Na_2_TeO_3_, Na_2_SeO_3_ or Na_2_SeO_4_) and oxacillin at the concentrations employed in the microbiological assays (0.25 mM and 0.2 µg/mL for the *S. aureus* ATCC 25923 strain, 0.5 mM and 150 µg/mL for the *S. aureus* MRSA HEMSA 5 strain or 0.5 mM and 2400 µg/mL for the *S. aureus* MRSA HEMSA 5M strain, respectively).

Next, 10 µM (final concentration) of DCHFA solubilized in ethanol (a 2 mM stock was prepared prior in ethanol and kept at −20 °C in the dark until further use) were added to the bacterial cultures and incubated at room temperature in the dark for 1 h. The samples were subjected to fluorescence spectrophotometric analysis. The fluorescence intensity was measured at 5 min intervals over a 60 min period using a microplate reader (EnSpire, PerkinElmer, Waltham, MA, USA), with an excitation wavelength of λ_ex_ = 480 nm and an emission wavelength of λ_em_ = 525 nm.

### 3.4. Inhibition of the Proteasome of S. Cerevisiae

Treatment of yeast was carried out according to the method reported recently by Letavayová *et al.* [49]. Briefly, *S. cerevisiae* was placed on Yeast Extract-Peptone-Dextrose medium (YPD medium) at 37 °C overnight. Inocula were prepared by suspending colonies of these cultures in fresh YPD broth until the cell suspension reached a density of 2 × 10^7^ cells/mL. Yeast cells were then incubated with 1000 μM of compounds at room temperature during 3 h under constant shaking [49]. By the end of the treatment, cells were collected by centrifugation and washed twice with phosphate buffered saline (PBS).

Cell pellets were resuspended in extraction buffer containing 50 mM Tris-HCl, 150 mM NaCl, 1 mM EGTA, 10% (*v*/*v*) glycerol, 0.5% (*v*/*v*) Nonidet P-40 substituent, and 1 mM MgCl_2_ (pH 7.5). The cells were lysed by sonication followed by 25 min incubation on ice and centrifugation at 15,000 *g* for 30 min. The total protein content in the supernatant was then assessed by the Bradford assay. 

Proteasomal activity was determined in cell extracts by incubating aliquots of total cellular protein (25–50 μg) at 37 ± 1 °C with the following fluorogenic substrates: suc-Leu-Leu-Val-Tyr-AMC (suc-LLVY-AMC; indicative of chymotrypsin-like activity) and z-Ala-Arg-Arg-AMC (z-ARR-AMC; indicative of trypsin-like activity). Fluorescence was recorded for 45 min (excitation wavelength of λ_ex_ = 380 nm and an emission wavelength of λ_em_ = 460 nm).

In each individual experiment, the control (untreated sample) was set at 100%. Final data represents the average of three independent experiments. Results were expressed as mean ± SD and the statistical significance was determined by one-way ANOVA followed by Bonferroni’s multiple comparison test. A value of *p* ≤ 0.05 was considered as statistically significant.

## 4. Conclusions

Eventually, we have been able to demonstrate that different selenium and tellurium compounds, ranging from selenite used as a selenium food supplement to multi-redox center organic molecules, are biologically active, also against some rather serious pathogens (such as two strains of MRSA). These compounds can either act on their own (such as compounds **1**–**4**) or in concert with traditional antibiotics. The role of selenite and other selenium supplements in fighting bacterial infections is therefore of considerable interest, especially since those compounds are also known to stimulate the human immune defense and to increase the antioxidant capacity—both highly beneficial processes during an infection.

Likewise, the biological chemistry of such compounds is truly fascinating and may combine different aspects of redox modulation and bioreductive formation of nanoparticles. No doubt, the relevant cellular targets, events, processes and subsequent biological activities certainly need to be studied in considerable more detail in the future. Here, the emerging selective fluorescent staining, quantification and microscopy techniques, together with classic activity assays and Western Blots will enable us to perform the kind of redox-related “intracellular diagnostics” which is now required to further illuminate the underlying cellular targets and processes [3]. Eventually, such knowledge may enable us to employ selenium, in its various chemical compositions and forms, effectively, in medicine and perhaps even in agriculture, and in the long run to enjoy fully probiotic drinks laced with natural selenium nanoparticles [50].
